# Innate‐like CD8+ T‐cells and NK cells: converging functions and phenotypes

**DOI:** 10.1111/imm.12925

**Published:** 2018-04-11

**Authors:** Ayako Kurioka, Paul Klenerman, Christian B. Willberg

**Affiliations:** ^1^ Nuffield Department of Medicine Peter Medawar Building for Pathogen Research University of Oxford Oxford UK; ^2^ NIHR Biomedical Research Centre Translational Gastroenterology Unit John Radcliffe Hospital Oxford UK

**Keywords:** CD8+ T‐cell, innate‐like T‐cell, mucosal‐associated invariant T‐cell, NK cell

## Abstract

New data in the worlds of both innate‐like CD8+ T‐cells and natural killer (NK) cells have, in parallel, clarified some of the phenotypes of these cells and also their associated functions. While these cells are typically viewed entirely separately, the emerging innate functions of T‐cells and, similarly, the adaptive functions of NK cells suggest that many behaviours can be considered in parallel. In this review we compare the innate functions of CD8+ T‐cells (especially mucosal‐associated invariant T‐cells) and those of NK cells, and how these relate to expression of phenotypic markers, especially CD161 and CD56.

**Abbreviations**
ADCC: antibody‐dependent cellular cytotoxicity
CMV: CYTOMEGALOVIRUS
IFN: interferon
ILC: innate lymphoid cell
KIR: killer immunoglobulin‐like receptors
MAIT cell: mucosal‐associated invariant T‐cell
MR1: MHC‐class I‐related protein 1
NK cell: natural killer cell
PLZF: promyelocytic leukaemia zinc finger protein
RORγ: RAR‐related orphan receptor gamma
TCR: T‐cell receptor
TRAIL: tumour necrosis factor‐related apoptosis‐inducing ligand
UCB: umbilical cord blood

## Introduction

Natural killer (NK) cells belong to the innate lymphoid cell (ILC) family, which share a dependency on the common cytokine receptor *γ* chain and the transcriptional repressor, inhibitor of DNA binding 2 (Id2) for their development.^1^, ^2^ These cells lack somatically rearranged antigen receptors like T‐ and B‐cell receptors, and are able to rapidly respond to microbial products, cytokine stimulation and contact with other leukocytes.^3^ NK cells form a distinct lineage of the family of ILCs and are often described as the cytotoxic arm of ILCs, or the innate counterpart of CD8+ T‐cells.^4^, ^5^, ^6^, ^7^, ^8^

CD8+ T‐cells have traditionally been studied in the context of their memory status, whether they are naïve or memory. However, in recent years a number, a large fraction of the human CD8+ T‐cell population has been identified as mucosal‐associated invariant T‐cells (MAIT cells)^9^, ^10^, ^11^; an innate‐like T‐cell population that is classically defined by its expression of a semi‐invariant T‐cell receptor (TCR), V*α*7·2‐J*α*33, and restriction by the major histocompatibility complex (MHC) class Ib molecule, MHC‐class I‐related protein 1 (MR1).^12^, ^13^ These cells share a functional phenotype with a family of other CD8+ T‐cells that express high levels of the C‐type lectin‐like receptor CD161.^14^ CD161 expression divides the CD8+ T‐cell population into three distinct functional subsets.^15^, ^16^ These subsets show varying degrees of innate (or NK‐like) activity according to their CD161 expression – thus CD161 expression can act as a marker of ‘innateness’ in T‐cell populations.

Recent insights into these subsets of CD8+ T‐cells as well as subsets of NK cells have highlighted interesting similarities between these populations. In this review, we will discuss what is currently known about each of the subsets of NK cells and CD8+ T‐cells, using the segregation of CD8+ T‐cells through CD161 expression as a starting point. We will, through this specific prism, explore shared functional features between these populations, as an alternative way of viewing these innate and adaptive counterparts. While there are many ways to approach this comparison through different individual surface markers, the consistent nature of the CD161‐associated functional effect in T‐cells, and the set of co‐expressed molecules that track with it, make this a simple and attractive focus.

## The ILC family

Three groups of ILCs have been defined with distinct patterns of cytokine production that mirrors that of T helper cell subsets, called group 1 ILCs (ILC1), group 2 ILC (ILC2) and group 3 ILC (ILC3).^17^ ILC2s produce IL‐5 and IL‐13 in response to epithelial cell‐derived IL33 and/or IL‐25.^18^, ^19^, ^20^ Their production of IL‐13 is critical for helminth expulsion,^18^, ^20^, ^21^ while they can also drive atopic dermatitis and allergic inflammations.^22^, ^23^ ILC3s in turn are characterized by their innate ability to secrete IL‐17A, IL‐17F, IL‐22 and GM‐CSF.^4^, ^24^ IL‐22 production by ILC3s is critical in containing commensal bacteria,^25^ and IL‐23‐responsive ILC3s also mediate colitis^26^ and accumulate in the inflamed intestine of CD patients.^27^ ILC3s also include the LTi cells involved in organizing tertiary lymphoid structures and tissue repair.^28^, ^29^

Group 1 ILCs were initially proposed to include NK cells and non‐NK cells, which secrete interferon (IFN)*γ*, and express T‐bet.^30^, ^31^ ILC1 cells are important anti‐bacterial cells, involved for example in the protection against *Salmonella enterica*.^32^ Furthermore, ILC1s accumulate in the inflamed intestine of CD patients.^30^ There also seems to be some plasticity between ILC1 and ILC3 cells.^32^, ^33^ Thus, ILCs secreting both IFN*γ* and IL‐17 in response to IL‐23, or IFN*γ* and IL‐22 in response to IL‐12+IL‐18 have been reported.^26^, ^34^ It is thought that both NK cells and ILC1s depend on IL‐15 for their development,^35^, ^36^, ^37^, ^38^ which is in contrast to ILC2s and ILC3s, which rely on IL‐7 and are depleted in IL‐7R*α* ^−/−^ mice.^19^, ^39^

### NK cells as CD8 T‐cell counterparts

More recently, NK cells have been shown to be a distinct lineage from the helper ILC subsets, as an Id2+ common progenitor was identified that gave rise to all helper‐like ILC subsets, including ILC1 cells, but not conventional NK cells.^38^ Thus, NK cells have been suggested to constitute the cytotoxic arm of ILCs, the innate counterpart of CD8+ T‐cells.^4^, ^5^, ^6^, ^7^, ^8^

Of note, the classification of helper ILC versus killer ILC is not black and white. (Similarly, the functional distinction between helper CD4+ T‐cells and killer CD8+ T‐cells may also be blurred.) The lack of a clear classification for ILCs is partly due to the cytotoxic potential of ILCs being relatively unknown.^40^ For instance, liver ILC1s express GrA and GrC at a higher level than liver NK cells,^41^ and show cytotoxic capacity.^42^ ILC1 cytotoxicity can also be mediated by tumour necrosis factor‐related apoptosis‐inducing ligand (TRAIL).^43^ However, transcriptional profiling of the murine ILC family has demonstrated that ILC1s and NK cells are distinguished by Eomes expression.^38^, ^41^ This is consistent with the dependence of NK cell maturation on Eomes.^44^ Further separating ILCs from conventional NK cells is found in the requirement for T‐bet. ILC1 cells require T‐bet for their development, and ILC3s require T‐bet to differentiate into NKp46+ ILC3s;^38^ however, NK cells can develop in the absence of T‐bet due to the redundancy between T‐bet and Eomes.^38^, ^44^, ^45^ CD8+ T‐cell development is similarly dependent on both T‐bet and Eomes, acting redundantly to induce effector functions.^46^

## Subsets of NK cells

### CD56^bright^ and CD56^dim^ NK cells

Two NK cell subsets have been well characterized in human peripheral blood, based on the expression level of CD56: CD56^bright^ and CD56^dim^ NK cells. The majority of peripheral blood NK cells are CD56^dim^ NK cells, while the ratio is inverted in most organs including the lymphoid tissues where the CD56^bright^ NK cells dominate,^47^ particularly due to the presence of tissue‐resident CD56^bright^ NK cells (as discussed later). CD56^bright^ NK cells are often described as the immunoregulatory population, based on their ability to secrete cytokines including IFN*γ*, and are considered to be poorly cytotoxic.^48^, ^49^ CD56^dim^ NK cells, on the other hand, have a highly cytotoxic phenotype and can efficiently lyse virus‐infected and tumour cell lines without prior activation.^50^, ^51^ However, this dichotomy based on cytokine production and cytotoxicity may be too simplistic, as CD56^bright^ NK cells can also be cytotoxic,^50^, ^51^, ^52^ while CD56^dim^ NK cells can also produce abundant cytokines and chemokines following activation.

Alternatively, these subsets can be functionally divided based on their responsiveness to specific signals. CD56^bright^ NK cells express high levels of cytokine receptors, which enables these cells to produce abundant IFN*γ* and proliferate in response to cytokines such as IL‐2, IL‐15, IL‐12, IL‐18 and IFN*α*.^48^, ^53^ As CD56^bright^ NK cells are the dominant NK cell population in tissues including lymph nodes and inflammatory sites, this allows them to interact with DCs and T‐cells at these sites.^54^, ^55^, ^56^, ^57^ In contrast, CD56^bright^ NK cells secrete little IFN*γ* in response to target cell recognition mediated by receptors such as NKG2D, even though the receptor is equally expressed by CD56^bright^ and CD56^dim^ cells.^58^ In turn, CD56^dim^ NK cells are the earliest and dominant IFN+ cells in response to activating receptor ligation.^58^, ^59^ Furthermore, CD56^dim^ NK cells are able to form more conjugates with infected or transformed cells,^60^, ^61^ and the expression of low‐affinity receptor III (CD16) is largely restricted to CD56^dim^ NK cells. These features, together with high expression of cytolytic molecules, allow CD56^dim^ NK cells to efficiently lyse target cells either directly or indirectly through CD16‐mediated antibody‐dependent cellular cytotoxicity (ADCC). The expression of a family of receptors called killer immunoglobulin‐like receptors (KIRs), which modulate the responsiveness of NK cells to activating receptor ligation,^62^, ^63^ is also restricted to CD56^dim^ NK cells.

Traditionally it has been thought that there is a linear developmental relationship between CD56^bright^ and CD56^dim^ NK cells. This is supported by studies showing that CD56^bright^ NK cells have longer telomeres.^64^ As murine NK cells do not express CD56, RAG2^−/−^ *γ*c^−/−^ mice transplanted with human haematopoietic stem cells have been used to show that CD56^bright^ NK cells differentiated linearly into CD56^dim^ NK cells, acquiring CD16 and KIR expression.^65^, ^66^ This has also been shown *in vitro* by culturing CD56^bright^ NK cells in the presence of synovial or skin fibroblasts, or cytokines.^52^, ^67^

Recent evidence from rhesus macaques, however, has suggested that the lineage origin of macaque NK cell homologues of CD56^bright^ NK cells (CD56+ CD16−) may be different from CD56^dim^ homologues (CD56 −CD16+).^68^ Furthermore, patients with mutations in the GATA2 gene lead to the absence of CD56^bright^ NK cells while CD56^dim^ NK cells are preserved.^69^, ^70^ Thus, whether CD56^bright^ and CD56^dim^ NK cells should be considered cells with independent lineages needs to be re‐examined.

### ‘Adaptive’ CD56^dim^ NK cells

Recently, a terminally differentiated population of NK cells with memory‐like properties has been described in the context of CMV.^71^, ^72^, ^73^ Primary MCMV infection has been shown to induce the clonal expansion of NK cells expressing the Ly49H receptor, which interacts with the m157 protein of MCMV, which persist in tissues for months after infection and, upon re‐challenge, undergo secondary expansion with enhanced effector functions.^72^ These NK cells thus exhibit memory‐like properties that were previously only attributed to cells of the adaptive immune system.

In humans, CMV infections are asymptomatic in healthy individuals, but immunosuppressed individuals, such as patients with human immunodeficiency virus (HIV), are at high risk of developing disease. CMV also skews the NK cell receptor repertoire in humans, with cells expressing the activating heterodimer NKG2C/CD94 expanding in recipients of solid organ^74^ or umbilical cord blood (UCB) transplantation^75^ during primary CMV infection or reactivation. These cells have an enhanced ability to secrete IFN*γ* in response to target cells or, even more so, upon CMV reactivation.^74^, ^75^, ^76^ Therefore, it has been suggested that these cells represent the human counterparts of Ly49H+ NK cells with memory‐like properties. These NKG2C+ cells can be identified by their high expression of CD57, and they express inhibitory KIRs specific for self‐MHC class I molecules.^74^, ^77^, ^78^

## Subsets of CD8+ T‐cells

### MAIT cells and CD161‐expressing CD8+ T‐cells

Mucosal‐associated invariant T‐cells were first identified in 1993 by virtue of their expression of a unique TCR *α* rearrangement,^79^ which was subsequently ascribed to a novel subset of T‐cells restricted by MR1.^80^ MAIT cells have been shown to detect a variety of microbes through the recognition of vitamin B metabolites presented by MR1.^12^, ^81^, ^82^ Furthermore, these cells were found to largely overlap with a unique subset of CD8+ T‐cells expressing CD161 at a high level, CD161++ CD8+ T‐cells.^11^, ^15^

Human peripheral blood CD8+ T‐cells can be divided into three distinct populations according to their relative expression level of the C‐type lectin‐like receptor CD161.^15^ These subsets are clearly present at birth, suggesting that they are distinct populations with pre‐programmed functional differences. MAIT cells are only a small fraction of the CD161++ CD8+ T‐cell population in cord blood,^83^ but slowly expand with age, making up more than 90% of the CD161++ CD8+ T‐cell population in adults.^11^, ^14^ Both MAIT and non‐MAIT CD161++ CD8+ T‐cells share functional features, most notably the ability to respond to innate cytokines in the absence of TCR stimulation, due to their high expression of cytokine receptors such as IL‐18R and IL‐12R.^9^, ^84^ This function is likely driven by the shared expression by MAIT and non‐MAIT CD161++ CD8+ T‐cells of the master transcription factor promyelocytic leukaemia zinc finger protein (PLZF).^14^, ^85^

Cells expressing CD161 at an intermediate level, CD161^+/int^ CD8+ T‐cells, also have a distinct pre‐programmed transcriptional profile that is shared between cord blood cells and their adult counterparts.^16^ The majority (96%) of these polyclonal cells in adults are memory cells, containing both effector memory and terminally differentiated memory cells as defined by CCR7 and CD45RA co‐expression. They constitutively express the cytotoxic mediators granzyme B and perforin. In contrast, a quarter of the cells that lack CD161‐expression, the CD161‐ CD8+ T‐cell population, are naïve CD8+ T‐cells, and even within the memory population express a lower level of granzyme B and perforin compared with the CD161^+/int^ CD8+ T‐cell population. The ability of a CD8+ T‐cell to respond to innate cytokines is correlated with the expression level of CD161, where it is only weakly associated with the CD161^+/int^ CD8+ T‐cell population and almost negligible in the CD161− CD8+ T‐cells.^14^

## Functional similarities between NK cell and CD8+ T‐cell subsets

### Cytokine responsiveness and proliferation

**Figure 1:**
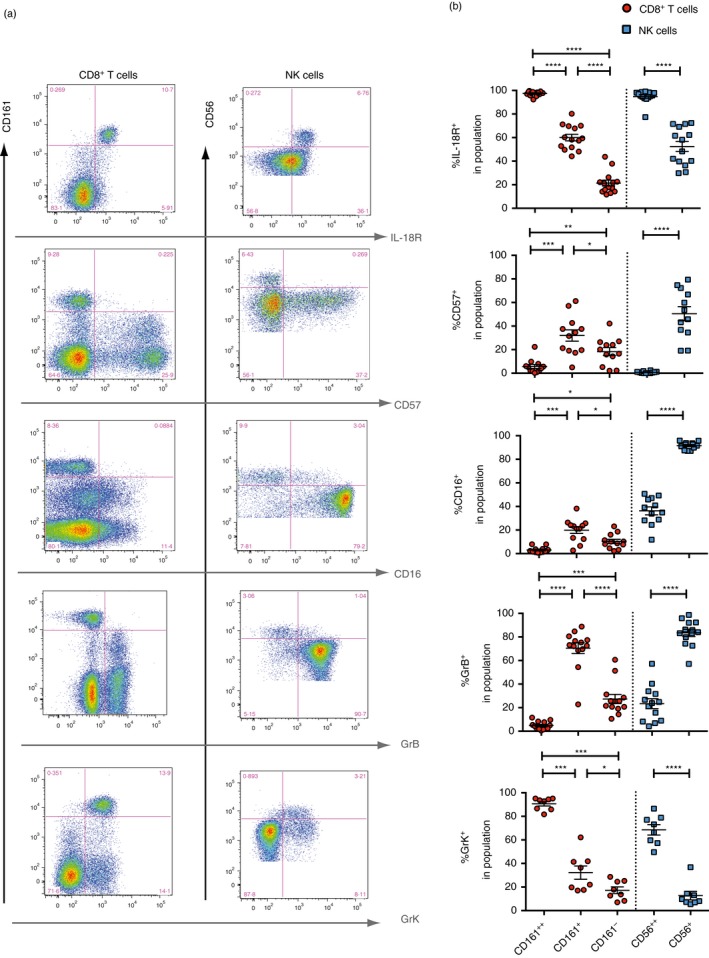
Shared phenotypic similarities between subsets of CD8+ T‐cells and natural killer (NK) cells. (a) Representative flow cytometry staining showing expression of various markers against CD161 expression on CD8+ T‐cells (left), or against CD56 expression on NK cells (right). Each panel shows the expression of the indicated marker within the same healthy donor, gated on CD8+ T‐cells or NK cells. (b) Frequencies of cells expressing the indicated marker within the CD161++, CD161+ and CD161‐ CD8+ T‐cells (red), within the CD56bright (CD56++) and CD56dim (CD56+) NK cells (blue), in 8–14 healthy individuals. ****P < 0·0001, ***P < 0·001, **P < 0·01, *P < 0·05 by repeated‐measures one‐way anova with Tukey's multiple comparisons test. All other comparisons were non‐significant.

CD56^bright^ NK cells and CD161++ CD8+ /MAIT cells both constitutive expression high levels of cytokine receptors, such as IL‐7R (CD127)^86^ and IL‐18R (Fig. 1). IL18R signalling induces the secretion of IFN*γ* in response to IL‐12+ IL‐18, compared with their respective ‘dim’ counterparts. CD161++ CD8+ T‐cells are uniformly high in IL‐18R expression regardless of whether they are MAIT cells or non‐MAIT, CD161++ V*α*7·2−, cells,^14^ and this is already pre‐programmed in fetal and cord blood cells.^83^, ^87^ Additionally, CD56^bright^ NK cells are known for their proliferative capacity in response to cytokines;^88^ for example, liver‐resident CD49a+ NK cells have been found to express high levels of CD25, and were able to proliferate in response to low doses of IL‐2.^89^ CD161++ CD8+ T‐cells are also highly proliferative upon stimulation with IL‐12 and IL‐15 compared with the CD161+ CD8+ T‐cell counterparts.^90^, ^91^

In turn, CD161++ CD8+ T‐cells have been described to be hyporesponsive to TCR signalling compared with their CD161+ and CD161− CD8+ T‐cell counterparts.^91^ Indeed, MAIT cells did not proliferate in response to PHA, which cross‐links the TCR/CD3 complex, but proliferated extensively when TCR stimulation was supplemented with anti‐CD28 and anti‐CD2, or with cytokines such as IL‐12 and IL‐18 during *Escherichia coli‐*induced proliferation. CD56^dim^ NK cells also have a heightened ability to respond to the cross‐linking of activating receptors such as NKp30 and CD16.^59^, ^66^ In particular, the terminally differentiated CD57++ NKG2C+ CD56^dim^ NK cells proliferate extensively in response to ligation of activation receptors such as NKG2C, and have a heightened capacity to perform ADCC through CD16.^92^ Thus, both CD161++ CD8+ T‐cells and CD56^bright^ NK cells have high cytokine responsiveness compared with other CD8+ and NK cell subsets, which is associated with a comparatively lower ability to respond to activating receptors alone.

**Figure 2:**
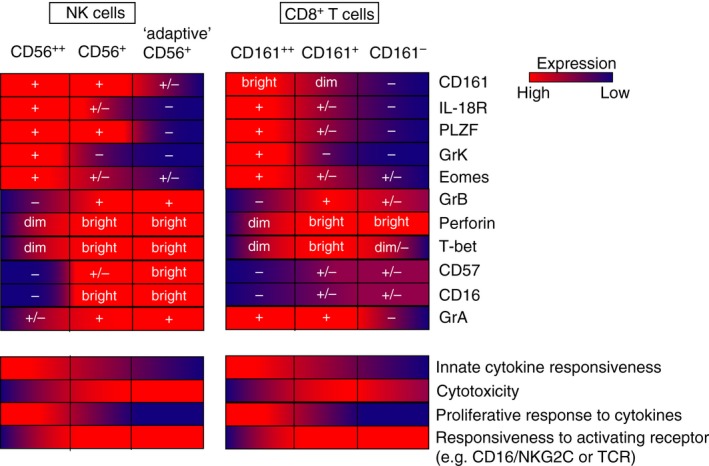
Model highlighting functional similarities between CD8+ T‐cell and natural killer (NK) cell subsets. The ability to respond to cytokines by secreting IFN ^©^ and proliferating inversely correlates with cytoxic potential at rest and responsiveness to activating receptor ligation in both CD8+ T‐cells and NK cells. The markers and transcription factors correlated with these functional properties are highlighted (top). ‘Adaptive’ CD56dim = terminally differentiated CD56dim NK cells. Bright/dim indicate expression levels, + and − signs indicate whether the population is mostly positive or negative for the marker indicated. Both ‘bright’ populations are also enriched in tissues and inflammatory sites where they may be activated by cytokines.

One of the key transcription factors regulating the cytokine responsiveness of both NK cells and T‐cells is thought to be PLZF. PLZF is critical for the function of innate‐like T‐cells, including MAIT cells,^85^ *γδ* T‐cells^93^ and iNKT cells.^94^, ^95^ Furthermore, newly described murine innate T‐cells such as the PLZF+ ROR*γ*t+ natural Th17 cells^96^ and PLZF+ T‐CD4 T‐cells^97^ also express PLZF, and are highly responsive to cytokines. CMV‐specific terminally differentiated NK cells, or ‘adaptive’ NK cells, have extensively downregulated PLZF expression, due to hypermethylation of the *ZBTB16* intronic sequence.^98^ PLZF was shown to directly bind the promoters of genes encoding signalling adaptor molecules, such as the SAP family receptor adaptor molecule EAT‐2, as well as CD161, IL‐12R and IL‐18R.^98^ The regulation of signalling adaptor molecules by PLZF not only affects cytokine responsiveness, but also responsiveness to receptor stimulation, as adaptive NK cells show a heightened ability to respond to ligation of receptors such as CD16 and NKG2C,^70^, ^98^ while NK cells from a PLZF‐deficient patient showed increased responsiveness of activating receptor ligation.^99^ Thus, PLZF appears to be a direct regulator of cytokine receptor expression and signalling proteins in both NK cells and T‐cells, and their differential expression level in subsets of these populations directly corresponds to their ‘innate‐ness’ (Fig. 2).

### Regulation of cytotoxicity

CD8+ T‐cells and NK cells constitute the cytotoxic arms of T‐cells and ILCs, respectively and, thus, share the expression of granzymes and perforin. Resting CD161++ CD8+ T‐cells, in particular the MAIT cells, express no GrB and little perforin, and instead are uniquely enriched for GrK expression.^90^ In turn, CD161+ CD8+ T‐cells are enriched for GrB and perforin expression, even compared with their CD161− CD8+ T‐cell counterparts.^16^ The GrK+ GrB− perforin^low^ phenotype of CD161++ CD8+ T‐cells was similar to the phenotype of CD56^bright^ NK cells (Fig. 1).^48^, ^100^ However, both CD56^bright^ NK cells^51^ and CD161++ CD8+ T‐cells (including the MAIT cells)^90^ can upregulate GrB and perforin, and become efficient killer cells, after activation. For example, the CD56^bright^ NK cell population can kill activated autologous CD4+ T‐cells in MS.^101^, ^102^ Furthermore, immature DCs are killed by CD56^bright^ NK cells in lymph nodes in a TRAIL‐dependent manner,^103^, ^104^ and IL‐2‐activated peripheral blood CD56^bright^ NK cells can become efficient killers.^50^, ^51^, ^52^ Similarly, CD69 + CXCR6 + tissue‐resident CD56^bright^ NK cells in lymphoid tissues have recently been shown to constitutively express perforin, but require pre‐activation to express GrB and become cytotoxic,^105^ much like MAIT cells.

Co‐expression of the transcription factors T‐bet and Eomes is critical for the cytotoxic functions of both CD8+ T‐cells and NK cells,^44^, ^106^ and the similarities in granzyme and perforin expression in these cells is reflected by their expression of the T‐bet and Eomes. Thus, both CD56^dim^ NK cells and CD161+ CD8+ T‐cells are enriched for Eomes+T‐bet^high^ cells,^16^, ^107^ while CD56^bright^ NK cells have an Eomes+T‐bet^low^ phenotype,^107^ which is mirrored by CD8+ MAIT cells.^108^ Of note, CD161+ CD8+ T‐cells express higher levels of GrB and perforin even compared with their CD161− CD8+ T‐cell counterparts, although both populations contain similar frequencies of effector memory T‐cells and terminally differentiated CD8+ T‐cells.^16^

### Enrichment in tissues

Perhaps the most interesting similarity between CD56^bright^ NK cells and CD161^bright^ CD8+ T‐cells is their enrichment in tissues.^56^

Although CD56^bright^ NK cells only constitute 10% of peripheral blood NK cells, they are enriched in tissues such as lymphoid tissues, stomach, adrenal gland, colorectal, liver and adipose tissues.^109^ Although some of these may include organ‐infiltrating NK cells, bona fide tissue‐resident CD56^bright^ NK cells have also recently been described (reviewed in^110^). These cells are retained in the tissues through their expression of CD69, chemokine receptors such as CXCR6 and CCR5, as well as adhesion molecules such as CD49a. For example, more than 75% of the NK cells in lymph nodes express CD56 at high levels,^52^, ^105^ the majority of which express CD69 and CXCR6, and have been shown to be lymphoid tissue‐resident.^105^ Similar enrichment of both tissue‐resident and circulating CD56^bright^ NK cells has been found in the spleen, bone marrow and tonsils. The liver is also enriched for NK cells, half of which have the CD56^bright^ phenotype.^111^ Liver‐resident NK cells have been described to be CD56^bright^ Eomes^high^, with high expression of CD69, CXCR6, CCR5,^112^, ^113^, ^114^ with Eomes^high^ cells retained in the human liver without recirculation for up to 13 years.^114^ Finally, it is known that more than 70% of lymphocytes in the uterine decidua during pregnancy are CD56^bright^ NK cells,^115^, ^116^ expressing adhesion markers such as CD49a, CD69 and CD103.

Mucosal‐associated invariant T‐cells are also known to be highly enriched in peripheral tissues, as reviewed elsewhere.^117^ In particular, they may make up to 30%–50% of all intrahepatic T‐cells, mediated by their expression of chemokine receptors such as CXCR6 and CCR6, which bind the chemokines CXCL16 and CCL20, respectively, that are constitutively expressed in the liver.^11^ Intrahepatic MAIT cells have been shown to be highly activated and express CD69, HLA‐DR and CD38, and are positioned around bile ducts within hepatic portal tracts.^111^, ^118^ Furthermore, as their name suggests, they are abundant within the gut, particularly in the jejunum and colon^10^, ^119^ through their expression of gut‐homing markers *α*4*β*7 and CCR9,^11^, ^13^, ^118^ as well as the lungs.^120^

In addition to their presence in tissues in the steady state, both CD161^bright^/MAIT and CD56^bright^ NK cell populations express chemokine receptors associated with inflammation such as CCR1, CCR5, as well as CCR6, which directs cells to sites of inflammation.^121^ Thus, CD56^bright^ NK cells are highly enriched and activated within synovial fluid from patients with psoriatic arthritis and rheumatoid arthritis (RA),^122^, ^123^ as well as in inflamed tissues in tuberculosis.^124^ Similarly, MAIT cells are also well established to be recruited to sites of inflammation,^81^, ^125^ and have been found to be enriched in the synovial fluid of patients with ankylosing spondylitis^126^ and RA^127^. They have also been shown to be recruited to the lung of patients with active tuberculosis infection,^81^, ^82^ increased in inflamed intestinal tissues in patients with inflammatory bowel disease,^125^ adipose tissues of obese patients,^128^ and are present in multiple sclerosis (MS) lesions.^129^, ^130^, ^131^ MAIT cell recruitment to inflamed tissues is further increased by their upregulation of chemokine receptors CXCR3, which binds IFN‐dependent ligands CXCL9/10/11 in inflamed tissues.^118^

## Lessons from NK cells to MAIT cells

A model summarizing the functional similarities between NK cell and CD8+ T‐cell subsets discussed in this review is shown in Fig. 2. This, of course, disregards the specificity of the TCR, the developmental relationship between the subsets, and many differences are not noted here. For instance, the type‐17 master transcription factor ROR*γ*t is expressed by MAIT cells^11^, ^128^ as well as non‐MAIT CD8+ T‐cells under inflammatory conditions,^132^, ^133^ but not in NK cells. However, the general inverse correlation between cytokine responsiveness and responsiveness to activating receptors may in part explain the functional similarities between CD8+ T‐cell and NK cell subsets. Furthermore, the tight regulation of cytotoxicity in both the CD56^bright^ NK cells and MAIT cells may be associated with their abundance in tissues and the need to limit tissue damage that may be caused by their non‐specific activation. These functions are associated with the shared expression of transcription factors that determines their effector functions. Interestingly, although these subsets of NK and T‐cells can be clearly delineated using markers such as CD56 and CD161, both of these markers are not directly involved in determining the differential functions of these subsets; in fact, CD161 has been suggested to have opposing functions on T‐ and NK cells.^134^

The similarities between NK cells and MAIT cells suggest that MAIT cells may share some functions already described in CD56^bright^ NK cells. For instance, the expansion of CD56^bright^ NK cells in patients with MS, receiving daclizumab treatment, is associated with better prognosis, due to their ability to kill pathogenic, highly activated CD4+ T‐cells.^102^ Cytotoxicity is mainly mediated through the expression of GrK^101^ and NKG2D.^135^ MAIT cells are also enriched in MS lesions and indeed early papers suggested that they play a regulatory role.^136^, ^137^ Whether MAIT cells are protective or pathogenic in MS may depend on their context of activation and the secretion of IL‐17, but the regulatory role for MAIT cells could be further examined in the context of NKG2D‐induced cytotoxicity. Additionally, it will be interesting to explore whether TRAIL‐induced cytotoxicity utilized by CD56^bright^ NK cells^103^, ^104^ may also be used by MAIT cells.

Innate‐like T‐cells are so‐called due to the fact that they share several functional features with NK cells. A recent study on iNKT cells demonstrated that iNKT cells share a broader transcriptional programme with NK cells than has been previously appreciated.^138^ In particular, many of the effector functions of innate‐like T‐cells can be shared with CD56^bright^ NK cells. Furthermore, terminally differentiated/adaptive NK cells have been shown to have epigenetic modifications resembling memory CD8+ T‐cells, particularly those affecting the expression of PLZF, IFN*γ*, cytokine receptors and signalling proteins.^98^, ^139^ Thus, comparison of the epigenetic signature between immature NK cells and innate‐like T‐cells may also reveal important similarities associated with their innate programming, such as the expression of other members of the BTB‐ZF protein family.

## Conclusions

Given the striking functional difference between CD56^bright^ and CD56^dim^ NK cells, defining which population is being investigated has been a requirement for NK cell research for a long time. However, bulk CD8+ T‐cell research generally only uses specific memory markers to differentiate the naïve and memory populations. Particularly with the advent of high‐dimensional analysis techniques, such as mass cytometry, inclusion of CD161 in panels, along with MAIT cell tetramers, may help to highlight true differences from differences accounted for by the variable frequency of MAIT cells and CD161++ T‐cells.

Overall, much effort in immunology has been focused on ‘splitting’ various subtypes, looking for differences in phenotype or function. There might be a reasonable argument made for the usefulness of ‘lumping’ cell types, i.e. looking for commonalities in phenotype and function. Because these cells do not work in isolation, such an integrative approach may help understand how responses to pathogens and tumours are mediated *in vivo* and how common pathways may be manipulated for patient benefit in disease.

## Disclosures

The authors have no conflicts of interest to declare.
